# Investigation of structural brain changes in Charles Bonnet Syndrome

**DOI:** 10.1016/j.nicl.2022.103041

**Published:** 2022-05-11

**Authors:** Michael J. Firbank, Katrina daSilva Morgan, Daniel Collerton, Greg J. Elder, Jehill Parikh, Kirsty Olsen, Julia Schumacher, Dominic ffytche, John-Paul Taylor

**Affiliations:** a Translational and Clinical Research Institute, Newcastle University, Newcastle upon Tyne, UK; b Northumbria Sleep Research, Department of Psychology, Faculty of Health and Life Sciences, Northumbria University, Newcastle upon Tyne, UK; c Department of Old Age Psychiatry, Institute of Psychiatry, King’s College London, UK

**Keywords:** Charles Bonnet Syndrome, Visual hallucinations, Eye disease, MRI

## Abstract

**Background and objectives:**

In Charles Bonnet Syndrome (CBS), visual hallucinations (VH) are experienced by people with sight loss due to eye disease or lesional damage to early visual pathways. The aim of this cross-sectional study was to investigate structural brain changes using magnetic resonance imaging (MRI) in CBS.

**Methods:**

Sixteen CBS patients, 17 with eye disease but no VH, and 19 normally sighted people took part. Participants were imaged on a 3T scanner, with 1 mm resolution T1 weighted structural imaging, and diffusion tensor imaging with 64 diffusion directions.

**Results:**

The three groups were well matched for age, sex and cognitive scores (MMSE). The two eye disease groups were matched on visual acuity. Compared to the sighted controls, we found reduced grey matter in the occipital cortex in both eye disease groups. We also found reductions of fractional anisotropy and increased diffusivity in widespread areas, including occipital tracts, the corpus callosum, and the anterior thalamic radiation. We did not find any significant differences between the eye disease participants with VH versus without VH, but did observe a negative association between hippocampal volume and VH severity in the CBS group.

**Discussion:**

Our findings suggest that although there are cortical and subcortical effects associated with sight loss, structural changes do not explain the occurrence of VHs. CBS may relate instead to connectivity or excitability changes in brain networks linked to vision.

## Highlights

• Reduced grey matter in the occipital cortex in eye disease groups.
• Widespread altered diffusivity in eye disease groups.
• No cortical or white matter changes associated with presence of visual hallucinations.
• Negative association between hippocampal volume and Hallucination severity.

## 1 Introduction

Charles Bonnet Syndrome (CBS) is a condition in which visual hallucinations (VH) are experienced by individuals following visual impairment due to eye disease or damage to visual pathways or cortex (O'Brien et al., 2020). The exact mechanism leading to these VH, and the predisposing factors for their occurrence in some, but not all people with visual impairment is not known, though it is hypothesised that following chronic visual deafferentation, the visual cortex becomes hyperexcitable leading to VH (Burke, 2002).

It is well established that there are structural brain changes in people with chronic visual impairment, particularly in the visual cortex (Nuzzi et al., 2020, Beer et al., 2020, Plank et al., 2011, Boucard et al., 2009). However, to what extent there are structural changes specific to CBS remains unknown. To date, only one report has investigated brain changes in a group of CBS patients. This looked specifically at cerebellar structures and found that CBS patients had reduced volume in cerebellar lobule VIII compared to eye disease participants without VH (Lawn and ffytche, 2021).

Visual hallucinations can be classed into two broad groups: (ffytche, 2007) simple (flashes of light, geometric patterns) and complex (hallucinations of people, animals, scenes). Simple hallucinations tend to be more common in eye disease (Urwyler et al., 2016, Cox and ffytche, 2014, Santhouse et al., 2000) but are not included in some definitions of CBS (ffytche, 2007). For the purposes of the present report, we are using the term CBS to include individuals with sight loss and either simple or complex visual hallucinations. It has been speculated that while the underlying mechanism is the same in both, simple hallucinations are the result of dysfunction in posterior occipital regions whilst complex hallucinations arise from dysfunction of higher cortical systems (ffytche et al., 2010).

Complex visual hallucinations are a frequent feature of Parkinson’s disease and Lewy body dementia (O'Brien et al., 2020), where they may be caused by a combination of visual and attentional deficits. Structural imaging studies in these conditions have found complex VH associated with reduced grey matter in occipital, inferior temporal and midline cortex (Pezzoli et al., 2021) with some reports of reduced hippocampal volume (Ibarretxe-Bilbao et al., 2008, Watanabe et al., 2013).

Hypotheses regarding the etiology of VH in neurodegenerative disease involve altered connections between visual regions (Esmaeeli et al., 2019, Tsukada et al., 2015, Shine et al., 2011). Supporting these hypotheses, alterations in white matter tracts, including the inferior and superior longitudinal fasciculus (which connect parieto-occipital cortex with anterior temporal and frontal cortices respectively) have been reported in Lewy body dementia patients with complex VH (Zorzi et al., 2021, Zarkali et al., 2020, Yuki et al., 2020).

The cholinergic system has also been implicated in the etiology of complex VH in neurodegenerative disease (O'Brien et al., 2020), with the main cortical innervation being from the nucleus basalis of Meynert (nbM). Reduced nbM volume has been shown in those with cognitive impairment with AD (Grothe et al., 2014), Lewy bodies (Schumacher et al., 2021) and also to be predictive of future cognitive decline in early Parkinson’s disease (PD) (Ray et al., 2018). Although preserved insight into the nature of hallucinations is needed for a diagnosis of CBS, there have been suggestions that impaired or fluctuating insight may be associated with, or predict, cognitive decline (Russell et al., 2018) or that prodromal Lewy body disease may be misdiagnosed as CBS (Terao, 2000).

The aim of this study was to compare people with CBS with two groups of similar age and sex: 1) people with eye disease who do not experience VH (Control-ED) and 2) people with normal vision without VH (Control). We aimed to investigate both cortical volume changes and differences in the white matter of the major visual pathways to establish whether the structural phenotype of neurodegenerative disease is also found in CBS.

We hypothesised that:

1) Participants with eye disease would have alterations in occipital grey matter volume and white matter integrity of visual pathways compared to participants with normal vision.
2) If complex VH in CBS have a shared mechanism with Lewy body disorders, then CBS participants would demonstrate reduced occipital, inferior temporal and hippocampal or nbM grey matter volume relative to ED participants without VH.
3) Similarly, if CBS has a shared mechanism with Lewy body disorders, reduced integrity of white matter tracts, particularly those connecting the occipital lobe would be found in CBS relative to ED participants without VH.
4) For both grey and white matter, changes would correlate with severity and frequency of VH, and that complex hallucinations would be associated with more widespread alterations extending beyond the occipital lobe to ventral occipito-temporal cortex compared to simple VH.
5) Cerebellar grey matter reduction is a specific feature of CBS vs ED participants without VH.

## 2 Methods

### 2.1 Participants

A total of 37 participants with eye disease were recruited (14 male; mean age 79) between June 2018 and November 2019. There were 18 Control-ED, and 19 people with CBS who had hallucinations continually or multiple times per week.

Participants from ED groups were identified via contact with consultants in ophthalmology, and from the Macular Society database of members interested in research participation. In addition, an advert was printed in the UK based Macular Society newsletter, providing contact details for members interested in participation. Control-ED participants were matched as closely as possible to the CBS group by age and visual acuity. Inclusion criteria included MMSE-blind > 24, and an absence of concurrent major psychiatric or neurological disease including any cause dementia. CBS patients met the diagnostic criteria of CBS of Teunisse et al. (1996) modified to include individuals with simple hallucinations.

The data presented here were taken from baseline assessment of participants in an intervention study (ISRCTN 16758036) and the number of participants was determined by an *a priori* power calculation for the intervention.

Data for participants without eye disease (Control) were taken from 19 healthy controls in previous studies investigating cognitive impairment (Firbank et al., 2021, Firbank et al., 2018). These were chosen to provide a good match of age and sex to the ED participants but with no significant loss of visual acuity or eye disease. They were scanned with exactly the same imaging sequences as the eye disease participants. Control participants with normal vision had no history of hallucinations in any modality. All study participants had full mental capacity and gave written informed consent.

Ethical approval was granted by the Tyne and Wear South Research Ethics Committee and Newcastle NHS Research and Development Committees (REC reference: 17/NE/0131). Screening and recruitment of participants occurred under the following NHS Foundation Trusts: Cumbria, Northumberland, Tyne and Wear (CNTW), Newcastle upon Tyne Hospitals (NuTH), City Hospitals Sunderland (CHSFT).

### 2.2 Assessment

Global visual acuity and contrast sensitivity (utilising both eyes) were evaluated using a computerised Freiburg visual acuity test, a standardised and reliable method for assessing visual function in visually impaired groups (Bach, 2007).

Detailed VH phenomenology was collected using an adapted version of the North East Visual Hallucinations Interview (NEVHI) (Mosimann et al., 2008). This asks about specific hallucination subtypes and collects details of temporal phenomenology (frequency and duration of each subtype) and a measure of distress / emotional impact. We also used an adapted hallucinations subscale from the Neuropsychiatric Inventory (Cummings et al., 1994). Although the NPI refers to hallucinations in all modalities, and typically requires a care-giver to complete (given its primary use in dementia patients), in the present study participants were directly asked about their experience and specifically about VH. Participants rated their VH in accordance using the NPI severity (0–3) and frequency (0–4) scales. As a measure of overall hallucination intensity, we calculated Severity × Frequency (0–12). We used the Mini Mental State Exam (MMSE) as a measure of global cognition. Control participants did the original MMSE (maximum score 30), whilst Control-ED and CBS participants performed the MMSE-blind (without pentagon, reading and writing a sentence) with a maximum score of 27.

### 2.3 Image acquisition

Participants were scanned on a 3 T whole body MR scanner (Achieva scanner; Philips Medical System, the Netherlands), with body coil transmission and an eight channel head coil receiver.

We acquired images including a whole brain structural 3D MPRAGE (magnetisation prepared gradient echo) scan with sagittal acquisition, slice thickness 1.0 mm, in plane resolution 1.0x1.0 mm; TR = 8.3 ms; TE = 4.6 ms; flip angle = 8°; SENSE factor = 2.

Diffusion tensor imaging acquisitions utilized a 2D spin-echo, echo planar imaging diffusion-weighted sequence with 59 slices: TR = 6100 ms; TE = 70 ms; flip angle = 90°; FOV = 270x270mm, pixel size = 2.1x2.1 mm; slice thickness = 2.1 mm; Diffusion-weighting was applied in 64 uniformly distributed directions (diffusion b = 1000 s.mm^−2^) and there were 6 acquisitions with no diffusion weighting (b = 0 s.mm^−2^). We also collected an identical image with b = 0 s.mm^−2^ but with the phase encoding direction reversed for distortion correction purposes.

### 2.4 Image processing

For analysis of grey matter volume, the T1 weighted structural images were segmented with the SPM12 (https://www.fil.ion.ucl.ac.uk/spm/) segment tool, then processed using the DARTEL (Diffeomorphic Anatomical Registration Through Exponentiated Lie algebra) Toolbox to create a group specific template, to which the individual images were spatially normalised. Grey and White matter segmentation images were modulated to preserve the total tissue amount during normalisation and smoothed with an 8 mm Gaussian filter for voxel based analysis, and with 1 mm for determining regional volumes. The template was then aligned with the MNI template using affine registration in SPM. Total volume of grey matter (GM), white matter (WM) and CSF were found using the ‘Tissue Volumes’ tool in SPM. Intracranial volume was calculated as the sum of all these. We also extracted the grey matter volume from structures in the regions V1-V5, hippocampus and basal forebrain (nbM) taken from the SPM Anatomy Toolbox (version 2.2c). Because previous studies found that patients with macular degeneration had localised grey matter loss in the posterior most portion of primary visual cortex, we divided V1 and V2 into anterior and posterior sections according to their intersection with the occipital pole region from the Neuromorphometrics atlas (http://neuromorphometrics.com/). The posterior sections of V1 and V2 were combined to create an occipital pole region (see eFigure 1). We used the SPM Anatomy toolbox and fslstats (https://fsl.fmrib.ox.ac.uk/fsl/fslwiki/) to find the mean pixel intensity of the modulated GM images (smoothed with 1 mm) multiplied by total ROI volume to give a measure of region volume per subject.

DTI data were processed using FSL (https://fsl.fmrib.ox.ac.uk/fsl/fslwiki) using the Topup program to correct susceptibility induced distortions using the two b = 0 s.mm^−2^ images with opposite phase encoding. The eddy package was then used to correct images for eddy current distortion, movement, and motion induced signal dropout. The FSL dtifit software was then used to calculate diffusion tensor images for each subject.

Based on previous evidence of improved tract alignment using tensor based vs FA based registration (Bach et al., 2014), with no benefit of tract-based skeleton projection (TBSS) when using improved registration (Schwarz et al., 2014), we used a voxel based analysis approach (Schwarz et al., 2014) with the dti-tk software (https://dti-tk.sourceforge.net/pmwiki/pmwiki.php) to register the diffusion tensor images from all participants to a study specific template (Zhang et al., 2007). Fractional anisotropy and mean diffusivity images were then calculated from the registered images, and smoothed with a Gaussian sigma of 1 mm using fslmaths. To exclude non WM areas, we created a mask with a threshold of 0.2 on the mean FA over all subjects. In order to label any significant regions using standard atlases, we used the FSL FNIRT software to coregister the template mean FA image with the JHU FA image provided in FSL. We then determined overlap of significant voxels with the binary JHU tract atlas JHU-ICBM-tracts-maxprob-thr0-1 mm (Hua et al., 2008) and JHU white matter labels. We also extracted mean FA from anterior thalamic radiation and optic radiation regions from voxels within the 0.2 threshold mask. The anterior thalamic radiation region was taken from the JHU atlas, and the optic radiation from the Juelich atlas (Eickhoff et al., 2005) as provided with FSL.

In addition, we used the FSL XTRACT software (Warrington et al., 2020) to identify and extract mean FA, MD and volume of key visual tracts [inferior longitudinal fasciculus (ILF), optic radiation (OR), vertical occipital fasciculus (VOF), inferior fronto-occipital fasciculus (IFO), and superior longitudinal fasciculus3 (SLF3)] for each subject. The total volume and mean FA and MD within each tract were calculated using the xtract_stats tool.

### 2.5 Statistics

For voxelwise comparisons of both GM and DTI, we used the general linear model in SPM, with three groups of sighted control, control-ED, and CBS with covariates of age, sex, and (for GM analysis) intracranial volume. We compared controls vs. all eye disease participants, and also looked within the CBS group between those with predominantly simple vs. complex hallucinations. Analyses within the eye disease group were also done controlling for visual acuity. Statistic images were thresholded voxelwise at p < 0.001 uncorrected, and then clusters which were significant after family wise error correction (p < 0.05) were reported. We used the SPM anatomy toolbox to identify the location of significant clusters.

R version 3.6.3 was used for all other statistical analyses. T-tests were done without assuming equal variance. Comparisons of regional volume and tract diffusion measurements between groups were done with a linear regression, with covariates of age, sex, intracranial volume, and group factor. There were no missing data for any of the variables under investigation.

To investigate association of region of interest data with VH intensity (NPI hallucination severity × frequency), we used linear regression to adjust the ROI data for age, sex and total intracranial volume, and then used Spearman rank correlation of the adjusted value against VH intensity.

### 2.6 Data availability

The data supporting the findings of this study are available on the basis of a formal data sharing agreement and depending upon data usage, agreement for formal collaboration and co-authorship, if appropriate.

## 3 Results

We excluded 4 participants (3 CBS, 1 Control-ED) due to severe white matter hyperintensities, leaving 33 participants with eye disease included in the analysis as shown in Table 1. eTable 1 provides details of VH phenomenology and eye disease. There were no significant differences between groups in age or sex, though the sighted controls had more years of education. The majority of patients had macular degeneration, and most (10/16) CBS participants experienced both simple and complex hallucinations (eTable 1), four had simple only, and two complex only. For analysis, we divided participants according to which VH type they primarily experienced, with 9/16 having predominantly complex VH, and 7/16 simple VH.

**Table 1:** Demographic details of the participants in the analysis.

	**CBS [N = 16]**	**Control-ED [N = 17]**	**Control [N = 19]**	**Stats**
Age	78.4 (10.6) [53:93]	78.4 (7.0) [67:87]	77.2 (7.0) [61:89]	F_2,49_ = 0.12 p = 0.89
Female gender	11/16 (68.75%)	11/17 (64.71%)	11/19 (57.89%)	p = 0.82
Years of education	12.0 (2.1) [10:15]	11.6 (2.2) [10:15]	14.7 (3.3) [11:24]	F_2,49_ = 7.49 p = 0.0015
MMSE %	96.5 (4.2) [88.9:100]	96.5 (4.2) [88.9:100]	96.0 (3.6) [90.0:100]	F_2,49_ = 0.12 p = 0.89
Years Since Diagnosis	12.0 (15.0) [1:61]	4.9 (7.8) [0.5:34]	–	t_20.4_ = 1.64 p = 0.12
Years of CBS	3.50 (3.41)	–	–	
Acuity	0.27 (0.29) [0.012:0.89]	0.38 (0.22) [0.10:0.68]	–	t_28.1_ = −1.18 p = 0.25
Contrast	50.2 (39.2) [1.0:100]	31.9 (35.3) [3.6:100]	–	t_24.5_ = 1.33 p = 0.20
Eye Disease (MD / GC / CT / RP / OT)	11/2/0/1/2	12/1/1/0/3	–	

Eye disease: MD = Macular Degeneration, GC = Glaucoma, CT = Cataracts, RP = Retinitis Pigmentosa, OT = other;

### 3.1 Hypothesis 1 – Eye disease vs. sighted participants

**Fig. 1:**
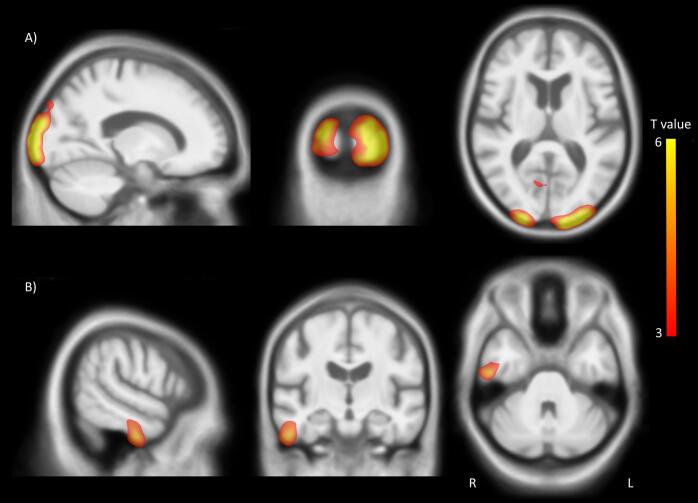
Significant clusters in the VBM analysis where grey matter in sighted participants is greater than the combined eye disease group. Threshold set at p = 0.001 voxelwise, uncorrected for multiple comparisons. T statistic colour bar shown.

In the VBM analysis of grey matter, we found regions of the visual cortex and right inferior temporal gyrus where grey matter density was lower in the combined eye disease group vs. sighted controls (see Fig. 1; Table 2).

**Table 2:** Significant clusters in the VBM analysis where grey matter in sighted participants is greater than the combined eye disease group.

**Cluster P**	**Cluster size (voxels)**	**x**	**y**	**z**	**Peak T**	
<0.001	6408	−24	−105	4	9.11	14% in V3d; 11% in V1; 11% in V2; 9% in V4lp; 6% in V3A; 5% in V3v
<0.001	2882	26	−102	10	6.55	16% in V2; 15% in V1; 9% in V3d; 9% in V3v; 4% in hOc4lp; 1% in V4(v)
0.003	1583	3	−75	−8	5.91	34% in V1; 21% in V3v; 15% in V2; 12% in V4(v); 4% in FG1
0.005	1444	56	−14	−36	5.24	Right inferior temporal gyrus

Threshold set at p = 0.001 voxelwise, uncorrected for multiple comparisons, then significant clusters with p < 0.05 FWE corrected reported. Labels are generated from the SPM Anatomy toolbox.

Similarly, as shown in Table 3, in the predefined regions of interest, there were reductions in occipital regions, particularly the occipital pole, but not hippocampus or nbM in eye disease vs sighted participants.

**Table 3:** Volumes of anatomical structures (mm^3^, mean (SD)) and p values for variables in linear regression.

	**CBS [N = 16]**	**Control-ED [N = 17]**	**Control [N = 19]**	**CBS vs Control-ED** **P value**	**ED vs Control** **P value**	**Sex P value**	**Age P value**	**TIV P value**	**Overall Model**
TBV (litres)	0.937 (0.13)	0.920 (0.08)	0.990 (0.07)	0.61	0.082	0.58	<0.001 **	<0.001 **	F_5,46_ = 52.63
Occipital pole	3174 (1065)	3179 (374)	4344 (519)	0.78	<0.001 **	0.16	0.32	<0.001**	F_5,46_ = 25.74
V1_ant	7073 (1187)	7094 (836)	7878 (1036)	0.91	0.014 *	0.67	<0.001 **	0.7	F_5,46_ = 4.95
V2_ant	3421 (490)	3450 (334)	3811 (444)	0.79	0.011 *	0.90	0.013 *	0.423	F_5,46_ = 4.14
V3a	2002 (414)	1959 (285)	2422 (244)	0.77	<0.001 **	0.53	0.25	0.047*	F_5,46_ = 9.43
V3d	3517 (736)	3537 (451)	4211 (478)	0.77	<0.001 **	0.95	0.033*	0.037*	F_5,46_ = 8.05
V3v	4288 (829)	4418 (424)	5149 (589)	0.36	<0.001 **	0.38	<0.001 **	0.037*	F_5,46_ = 15.69
V4v	3757 (682)	3885 (380)	4259 (364)	0.32	0.005 *	0.30	<0.001 **	0.22	F_5,46_ = 10.14
hOc4la	5190 (962)	5298 (534)	5736 (626)	0.42	0.107	0.94	0.003*	0.003*	F_5,46_ = 9.52
hOc4lp	3719 (803)	3936 (461)	4527 (570)	0.18	<0.001 **	0.27	<0.001 **	0.07	F_5,46_ = 12.05
V5	504 (130)	519 (83)	574 (90)	0.58	0.063	0.49	<0.001 **	0.52	F_5,46_ = 4.55
Hippocampus	4426 (805)	4162 (565)	4680 (632)	0.22	0.181	0.68	<0.001 **	0.015*	F_5,46_ = 9.22
nbM	221 (38)	217 (24)	227 (25)	0.87	0.877	0.035*	0.001*	0.004*	F_5,46_ = 5.51

TIV = total intracranial volume. nbM = nucleus basalis of Meynert. Occipital pole is the intersection of regions V1 and V2 with the neuromorphic atlas occipital pole region. V1_ant and V2_ant are the V1 and V2 regions excluding the occipital pole area.

**Fig. 2:**
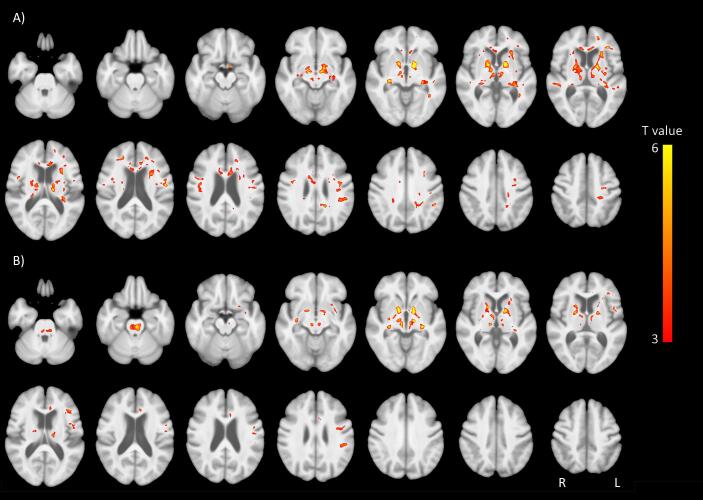
Diffusion analysis: Clusters of significantly altered A) FA, and B) MD in the visually impaired group compared to the normally sighted controls. Threshold set at p = 0.001 voxelwise, uncorrected for multiple comparisons. T statistic colour bar shown. Red indicates decreased FA and increased MD in the eye disease group. (For interpretation of the references to colour in this figure legend, the reader is referred to the web version of this article.)

**Fig. 3:**
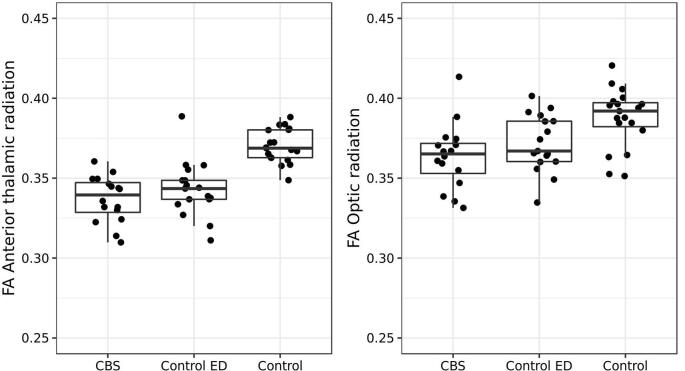
Mean FA within optic radiation and anterior thalamic radiation.

The voxel based analysis of diffusion parameters found widespread regions where FA was higher (Fig. 2, eTable 2) and MD lower (Fig. 2, eTable 3) in the group with normal vision compared to the eye disease patients, particularly in the anterior thalamic radiation, anterior commissure locale, and fornix/stria terminalis. Fig. 3 shows the mean FA within anterior thalamic radiation and optic radiation. We also investigated the relationship between duration of eye disease and diffusion changes (see eFigure 2). There was a significant association in the optic tract between FA and duration (t_29_ = −2.87; p = 0.008) & age (t_29_ = −2.3; p = 0.027), but this was driven by one individual with over 60 years of eye disease, and after removal of this datapoint, only the relationship with age (t_28_ = 2.35;p = 0.026) was significant.

The region of interest analysis of the visual tracts also found significant difference in FA (and to a lesser extent MD and tract volume) between normally sighted and ED participants (Table 4).

**Table 4:** DTI values of tracts and p values for variables in ANCOVA.

**Region**	**CBS N = 16**	**Control-ED N = 17**	**Control N = 19**	**CBS v Control-ED P value**	**ED vs Control P value**	**Age P value**	**Sex P value**	**TIV P value**	**Model Statistics**
ILF mean FA	0.331 (0.019)	0.334 (0.019)	0.344 (0.02)	0.68	0.05	0.011*	0.82		F_4,47_ = 2.97
OR mean FA	0.364 (0.024)	0.369 (0.025)	0.394 (0.016)	0.55	<0.001 **	0.024*	0.43		F_4,47_ = 6.70
VOF mean FA	0.264 (0.021)	0.272 (0.019)	0.29 (0.021)	0.27	<0.001 **	0.07	0.90		F_4,47_ = 4.68
IFOF mean FA	0.335 (0.02)	0.339 (0.019)	0.361 (0.016)	0.56	<0.001 **	0.008*	0.96		F_4,47_ = 7.83
SLF3 mean FA	0.282 (0.016)	0.283 (0.015)	0.31 (0.012)	0.95	<0.001 **	0.10	0.40		F_4,47_ = 12.71
ILF mean MD	892 (36)	891 (44)	880 (42)	0.90	0.391	<0.001 **	0.35		F_4,47_ = 5.08
OR mean MD	940 (49)	945 (54)	900 (58)	0.76	0.005 *	<0.001 **	0.67		F_4,47_ = 7.78
VOF mean MD	1019 (66)	1005 (63)	965 (74)	0.50	0.019 *	0.001*	0.45		F_4,47_ = 4.73
IFOF mean MD	925 (41)	924 (49)	893 (45)	0.93	0.007 *	<0.001 **	0.25		F_4,47_ = 10.32
SLF3 mean MD	1048 (57)	1046 (48)	1008 (59)	0.86	0.006 *	<0.001 **	0.007*		F_4,47_ = 7.04
ILF volume mm^3^	29,689 (3370)	28,333 (3400)	32,589 (3794)	0.34	0.008 *	0.66	0.69	0.016 *	F_5,46_ = 5.54
OR volume mm^3^	29,902 (3876)	29,560 (2477)	32,223 (2368)	0.94	0.036 *	0.81	0.36	0.006 *	F_5,46_ = 4.44
VOF volume mm^3^	18,256 (3069)	18,524 (2588)	17,254 (2137)	0.47	0.013 *	0.99	0.027 *	0.001 *	F_5,46_ = 3.27
IFOF volume mm^3^	50,040 (6290)	49,232 (5366)	54,349 (4732)	0.83	0.03 *	0.48	0.61	0.006 *	F_5,46_ = 5.43
SLF3 volume mm^3^	28,055 (4767)	28,903 (5597)	31,588 (4652)	0.51	0.108	0.78	0.33	0.117	F_5,46_ = 1.49

ILF = inferior longitudinal fasciculus, OR = optic radiation, VOF = vertical occipital fasciculus, IFOF = inferior fronto-occipital fasciculus, SLF3 = superior longitudinal fasciculus3, TIV = total intracranial volume.

### 3.2 Hypothesis 2 & 3 – Eye disease with vs. without visual hallucinations

We did not find any significant differences between the VH group and Control-ED in the VBM grey matter analysis, nor in any of the regions of interest (Table 3) in grey matter. This was still the case when we covaried for visual acuity and duration of eye disease (eTable 4). Similarly, we did not find any difference in white matter in either the VBM nor tract analysis (Table 4, eTable 5).

### 3.3 Hypothesis 4 – VH severity and complexity

In the regions of interest, we investigated the relationship between the VH intensity in the patient group and structural volumes, adjusting for age, sex and TIV. Hippocampal volume was associated with lower VH intensity scores (Spearman *rho* = −0.839, *P* < 0.001, significant with Bonferroni correction for 12 ROIs) (eFigure 3). To further characterise the association we looked at hippocampal relationship between VH severity and frequency separately, finding a significant association for severity (*rho* = −0.77, *P* < 0.001) but not frequency (*rho* = 0.44; *P* = 0.08). Neither hippocampal volume, nor VH intensity was significantly associated with MMSE. No other region of grey matter, nor any of the DTI white matter measures showed a significant relationship with VH intensity.

We did not find any significant differences in VBM or regional analyses (eTables 6 + 7) between those with primarily simple compared to complex visual hallucinations in either the grey or white matter.

### 3.4 Hypothesis 5 – VH and the cerebellum

**Fig. 4:**
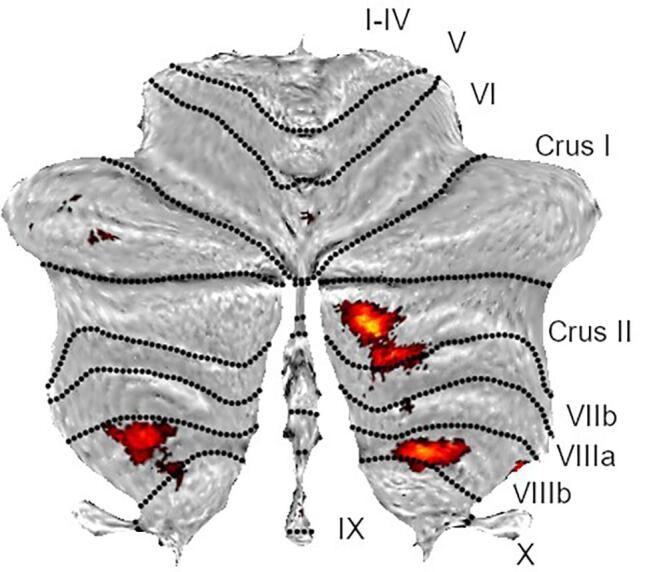
Clusters of significantly reduced cerebellar grey matter in those with predominantly simple vs complex hallucinations. Threshold set at p = 0.001 voxelwise, uncorrected for multiple comparisons.

In the analysis of cerebellar grey matter, we did not find any significant differences between the CBS and Control-ED group, although there were two clusters which were significant before correcting for multiple comparisons (MNI -30,-38,-49 cluster *P_uncorr_* = 0.025, in lobule VIIIa and VIIIb and 6-78,-21, cluster *P_uncorr_* = 0.004 right lobule VI). In the comparison of those with primarily complex vs. simple VH, there were clusters of decreased cerebellar grey matter (see Fig. 4, eTable 8) in those with simple hallucinations in right Lobule VIIa Crus II (MNI 18,-84,-41; cluster *P*_FWE_ < 0.001), and bilateral lobule VIIIb (left MNI -24,-50,-59; cluster *P*_FWE_ = 0.009, right MNI 16,-54,-55 *P_FWE_* = 0.056).

Since reduced grey matter in lobule VIIIb was found in those with VH in a recent report (Lawn and ffytche, 2021), we extracted the mean GM within the entire lobule, using the lobule atlas in SUIT. A 3 group ancova (Control-ED, simple, complex VH) with covariates of age, sex, total intracranial volume found a significant group difference (*F*_2,27_ = 4.60; *P* = 0.019) with Tukey post hoc test showing reduced grey matter volume in the simple group vs Control-ED (*P* = 0.036) and vs complex VH (*P* = 0.037), but no difference between the complex VH and Control-ED group (eFigure 4).

## 4 Discussion

As expected, we found that compared to sighted participants, those with eye disease had reduced occipital grey matter and widespread white matter alterations, particularly in the vicinity of the anterior commissure. We did not find any differences in the VH group compared to the control ED group in cortical grey matter regions or white matter tracts implicated in the underlying mechanism of VH in neurodegenerative disease. There were also no differences between those with predominantly simple vs complex hallucinations. However, we did find an association of increased VH severity with decreased hippocampal volume. There was also evidence of reduced cerebellar grey matter in lobule VIIa and VIIIb in those with predominantly simple VH.

Consistent with previous structural brain imaging studies of visual impairment (Nuzzi et al., 2020, Beer et al., 2020, Plank et al., 2011, Boucard et al., 2009), in the ED group we found marked reductions in grey matter in the occipital lobe, including most of the visual areas V1-V4. This was most pronounced in the occipital pole; the majority of our ED participants had macular degeneration, which primarily affects the central field of vision, which has cortical representation in this area (Plank et al., 2011, Boucard et al., 2009).

Also consistent with previous research, we found more extensive changes in the white matter than grey matter (Hernowo et al., 2014). Most previous studies using diffusion MRI in eye disease have focussed on specific tracts, rather than whole brain analysis, typically finding reduced tract integrity and volume in posterior visual pathways, including the inferior longitudinal fasciculus, inferior fronto-occipital fasciculus (Reislev et al., 2016), and the optic radiation (Yoshimine et al., 2018). A study of the thalamus and its connections (Reislev et al., 2016), found FA changes in the whole thalamus, and the thalamo-occipital & thalamo-temporal tracts of blind participants. Similar to our findings, a recent study using a tractography approach (Cavaliere et al., 2020) reported reduced FA in the anterior commissure fibres. Our study adds to these previous reports by showing widespread white matter changes occur in people with sight loss.

Although our ROI analysis demonstrated changes to occipital tracts, in the whole brain voxelwise analysis, we found more statistically significant changes in anterior than posterior regions of eye disease participants. This is different to some previous reports in younger ED cohorts where the more statistically significant changes are in posterior than anterior white matter (Olivio et al., 2015). In part this may be because of increased variability of diffusion metrics in the posterior vs anterior regions (see Fig. 3), which has also been reported in other studies of older individuals (Booth et al., 2013). Alternatively, it has been suggested that plasticity of the thalamocortical visual pathways is more pronounced in younger than older patients which could also contribute to the posterior predominance in younger patients (Olivio et al., 2015). Hernowo et al. (2014) looked at volume of grey and white matter in juvenile and age-related macular degeneration. Similar to our findings, they identified decrease in grey matter confined to the occipital lobe, but reduction in white matter volume throughout the brain, particularly in the frontal lobes of the AMD group, with juvenile MD white matter changes restricted to posterior regions. They speculated that this might be due to a link between AMD and Alzheimer’s disease. A recent review concluded that there is good evidence of an association between visual impairment and cognitive decline (Nagarajan et al., 2022) with shared vascular risk factors for both being a possible mechanism. It may be that changes in white matter outside the visual tract in older patients which we and others have observed are evidence of increased vascular disease or other neuropathology in eye disease. However, this does not explain the frontal predominance of the white matter changes or why they are also apparent in younger patients. Longitudinal studies of people with recently diagnosed eye disease across the age spectrum would help to clarify this issue.

Regarding structural imaging of the cortex in CBS, we are only aware of one case report (Martial et al., 2019), which found decreased grey matter in associative and multimodal cortices of an 85 year old with CBS compared to visually impaired controls. However, there was a substantial age difference between the CBS patient and the controls (mean age 53) which may have accounted for some of the difference. We are not aware of any studies using diffusion MRI in CBS.

Burke (2002) hypothesized that simple hallucinations in eye disease (e.g. flashes of light, regular patterns) were due to deafferentation of the visual cortex arising as a consequence of eye disease, leading to hyperexcitability of the primary visual cortex. CBS patients can experience complex VH (Urwyler et al., 2016) similar to those frequently experienced by people with Parkinson’s disease or cognitive impairment with Lewy bodies. A number of hypotheses have been suggested for these VH, involving dysfunctional communication of visual pathways, including the frontal lobe and thalamic radiation, as well as occipital tracts (Esmaeeli et al., 2019, Tsukada et al., 2015, Shine et al., 2011). An fMRI study of CBS experiencing hallucinations (ffytche et al., 1998) found increased activity in regions of extrastriate cortex relevant for the percept (i.e. hallucinations of faces were associated with activity in the fusiform face area) along with a generalised increase in ventral extrastriate activity between hallucination episodes.

We found grey matter reductions in primary and extrastriate visual cortex as well as extensive white matter changes in eye disease vs. sighted participants, consistent with loss due to deafferentation in ED and overlapping regions associated with VH in Lewy body disorders. However, our overall finding of no cortical grey matter or white matter differences between those with vs without VH, nor between simple vs complex hallucinations, suggest that the structural changes present in eye disease are not by themselves responsible for visual hallucinations. Instead, it seems likely that the changes allow VH, and that functional changes (for example, in terms of connectivity or cortical excitability) (daSilva et al., 2018) as the visual system adapts to reduced input and the resulting cortical loss due to under-stimulation of primary visual areas, lead to VH in some people. Future studies investigating functional connectivity in CBS using fMRI and Magnetoencephalography (MEG) would be of great value in understanding the condition. It may also be of benefit to compare CBS against other forms of sensory deprivation induced phenomena such as tinnitus or phantom limb pain.

In our study, we found a trend towards reduced grey matter in cerebellar lobule VIIIb in the overall VH group. This was pronounced in those with predominantly simple VH, as they had significantly lower GM in the VIIIb region compared to both those with complex VH and the control group. Lawn and ffytche (2021) found reduced GM in regions IX, VIIIa, VIIIb, and VIIb in a group containing both eye disease and PD participants, with a mixture of simple and complex VH. Reductions in glucose metabolism, but not cortical atrophy, in similar regions have been found in patients with DLB and VH using FDG-PET imaging (Zorzi et al., 2021). It may be that our finding of cerebellar volumetric reductions in simple, but not complex hallucinators was partially a result of the relatively small number of participants in our sub groups. Nevertheless, the VIIIb clusters are in an area of the cerebellum recently shown to be retinotopically organised (van Es et al., 2019), in the somatomotor network (Buckner et al., 2011) and activated in action observation (King et al., 2019) and so changes in this location are likely to impact visual processing and speculatively may relate to specific VH phenomenological features. The cerebellum is involved in saccadic eye motion, utilising sensory prediction errors to compare expected and observed input (Schlerf et al., 2012, Thier and Markandy, 2019) and is functionally connected to attention networks (Buckner et al., 2011) thought to underlie VH in PD. Alterations of low level visual input and incorrect prediction encoding or attentional dysfunction are features of several VH models (O'Brien et al., 2020). It is plausible that for both CBS and neurodegenerative conditions, incorrect tracking of the visual scene leads to discrepancies which are incorrectly interpreted by higher visual areas to result in VH.

We found associations between VH severity and hippocampal volume, but not with occipital volume. In a separate study which included people with CBS, reduced insight into VH was associated with both reduced MMSE score and increased VH distress (Montagnese et al., 2021). It may be that hippocampal volume is an indicator of neurodegenerative processes like AD, associated with cognitive decline. Perhaps reduced insight into the nature of VH, as a result of cognitive decline, might then cause VH to be perceived as more severe/distressing. However, our participants were relatively old and thus at risk of AD; younger people with CBS can also experience distressing VH, which is presumably not due to cognitive decline. Future work could investigate the associations between cognitive decline, insight and hallucination distress.

Strengths of our study include the well-characterised cohort with participants with and without eye disease, and additionally the sample size, as to our knowledge this is largest MRI study in CBS. Limitations are that our participants were relatively old, representative of the majority of patients with CBS but not those with younger onset CBS where neurodegenerative changes are less likely to be present. Although most of the patients with eye conditions had macular disease as their primary pathology, some had other eye diseases, either as primary or secondary pathology, and it is possible that this would increase the heterogeneity of brain changes, and the type of VH experienced.

In conclusion, in the eye disease group as a whole, we found grey matter reductions in occipital areas, and widespread changes to white matter tracts. We did not find any structural differences between the overall CBS patients and the control-ED group; however, we did find reduced grey matter in cerebellar lobules VIIa and VIIIb in those with simple relative to complex VH. Our data suggest that structural alterations may predispose to VH, but the primary mechanism in CBS is likely to be alterations in functional connectivity or cortical excitability as the visual system adapts to reduced input and concomitant cortical changes.

### CRediT authorship contribution statement

**Michael J. Firbank:** Writing – original draft, Formal analysis. **Katrina daSilva Morgan:** Data curation, Investigation, Writing – review & editing. **Daniel Collerton:** Conceptualization, Methodology, Writing – review & editing. **Greg J. Elder:** Funding acquisition, Conceptualization, Writing – review & editing. **Jehill Parikh:** Methodology, Writing – review & editing. **Kirsty Olsen:** Data curation, Investigation, Writing – review & editing. **Julia Schumacher:** Writing – review & editing. **Dominic ffytche:** Funding acquisition, Supervision, Conceptualization, Writing – review & editing. **John-Paul Taylor:** Funding acquisition, Supervision, Conceptualization, Writing – review & editing.

## Declaration of Competing Interest

The authors declare that they have no known competing financial interests or personal relationships that could have appeared to influence the work reported in this paper.

## References

[b0005] Bach M. (2007). The Freiburg visual acuity test - variability unchanged by post-hoc re-analysis. Graefes Arch. Clin. Exp. Ophthalmol..

[b0010] Bach M., Laun F.B., Leemans A. (2014). Methodological consideration on tract-based spatial statistics (TBSS). NeuroImage.

[b0015] Beer A.L., Plank T., Greenlee M.W. (2020). Aging and central vision loss: Relationship between the cortical macro-structure and micro-structure. NeuroImage.

[b0020] Booth T., Bastin M.E., Penke L. (2013). Brain white matter tract integrity and cognitive abilities in community-dwelling older people: the Lothian birth cohort 1936. Neuropsychology.

[b0025] Boucard C.C., Hernowo A.T., Maguire R.P. (2009). Changes in cortical grey matter density associated with long-standing retinal visual field defects. Brain.

[b0030] Buckner R.L., Krienen F.M., Castellanos A., Diaz J.C., Yeo B.T.T. (2011). The organization of the human cerebellum estimated by intrinsic functional connectivity. J. Neurophysiol..

[b0035] Burke W. (2002). The neural basis of Charles Bonnet hallucinations: a hypothesis. J. Neurol. Neurosurg. Psychiatry.

[b0040] Cavaliere C., Aiello M., Soddu A. (2020). Organization of the commissural fiber system in congenital and late-onset blindness. NeuroImage: Clin..

[b0045] Cox T.M., ffytche D.H. (2014). Negative outcome Charles Bonnet Syndrome. Br. J. Ophthalmol..

[b0050] Cummings J.L., Mega M., Gray K., Rosenberg-Thompson S., Carusi D.A., Gornbein J. (1994). The neuropsychiatric inventory: comprehensive assessment of psychopathology in dementia. Neurology.

[b0055] daSilva M.K., Elder G.J., ffytche D.H., Collerton D., Taylor J.P. (2018). The utility and application of electrophysiological methods in the study of visual hallucinations. Clin. Neurophysiol..

[b0060] Eickhoff S.B., Stephan K.E., Mohlberg H. (2005). A new SPM toolbox for combining probabilistic cytoarchitectonic maps and functional imaging data. NeuroImage.

[b0065] Esmaeeli S., Murphy K., Swords G.M., Ibrahim B.A., Brown J.W., Llano D.A. (2019). Visual hallucinations, thalamocortical physiology and Lewy body disease: a review. Neurosci. Biobehav. Rev..

[b0070] ffytche D.H. (2007). Visual hallucinatory syndromes: past, present, and future. Dialogues Clin. Neurosci..

[b0075] ffytche D.H., Howard R.J., Brammer M.J., David A., Woodruff P., Williams S. (1998). The anatomy of conscious vision: an fMRI study of visual hallucinations. Nat. Neurosci..

[b0080] ffytche D.H., Blom J.D., Catani M. (2010). Disorders of visual perception. J. Neurol. Neurosurg. Psychiatry.

[b0085] Firbank M.J., Parikh J., Murphy N. (2018). Reduced occipital GABA in Parkinson disease with visual hallucinations. Neurology.

[b0090] Firbank M.J., O'Brien J.T., Durcan R. (2021). Mild cognitive impairment with Lewy bodies: blood perfusion with arterial spin labelling. J. Neurol..

[b0095] Grothe M.J., Ewers M., Krause B., Heinsen H., Teipel S.J. (2014). Basal forebrain atrophy and cortical amyloid deposition in nondemented elderly subjects. Alzheimers Dement.

[b0100] Hernowo A.T., Prins D., Baseler H.A. (2014). Morphometric analyses of the visual pathways in macular degeneration. Cortex.

[b0105] Hua K., Zhang J., Wakana S. (2008). Tract probability maps in stereotaxic spaces: Analyses of white matter anatomy and tract-specific quantification. NeuroImage.

[b0110] Ibarretxe-Bilbao N., Ramírez-Ruiz B., Tolosa E. (2008). Hippocampal head atrophy predominance in Parkinson's disease with hallucinations and with dementia. J. Neurol..

[b0115] King M., Hernandez-Castillo C.R., Poldrack R.A., Ivry R.B., Diedrichsen J. (2019). Functional boundaries in the human cerebellum revealed by a multi-domain task battery. Nat. Neurosci..

[b0120] Lawn T., ffytche D. (2021). Cerebellar correlates of visual hallucinations in Parkinson's disease and Charles Bonnet syndrome. Cortex.

[b0125] Martial C., Larroque S.K., Cavaliere C. (2019). Resting-state functional connectivity and cortical thickness characterization of a patient with Charles Bonnet syndrome. PLoS ONE.

[b0130] Montagnese M., Vignando M., Collerton D. (2021). Cognition, hallucination severity and hallucination-specific insight in neurodegenerative disorders and eye disease. Cogn. Neuropsychiatry.

[b0135] Mosimann U.P., Collerton D., Dudley R. (2008). A semi-structured interview to assess visual hallucinations in older people. Int. J. Geriatr. Psychiatry.

[b0140] Nagarajan N., Assi L., Varadaraj V. (2022). Vision impairment and cognitive decline among older adults: a systematic review. BMJ Open.

[b0145] Nuzzi R., Dallorto L., Vitale A. (2020). Cerebral modifications and visual pathway reorganization in maculopathy: a systematic review. Front. Neurosci..

[b0150] O'Brien J., Taylor J.P., Ballard C. (2020). Visual hallucinations in neurological and ophthalmological disease: pathophysiology and management. J. Neurol. Neurosurg. Psychiatry.

[b0155] Olivio G., Melillo P., Cocozza S. (2015). Cerebral involvement in Stargardt's disease: a VBM and TBSS study. Vis. Neurosci..

[b0160] Pezzoli S., Sánchez-Valle R., Solanes A. (2021). Neuroanatomical and cognitive correlates of visual hallucinations in Parkinson's disease and dementia with Lewy bodies: voxel-based morphometry and neuropsychological meta-analyis. Neurosci. Biobehav. Rev..

[b0165] Plank T., Frolo J., Brandl-Rühle S. (2011). Grey matter alterations in visual cortex of patients with loss of central vision due to hereditary retinal dystrophies. NeuroImage.

[b0170] Ray N.J., Bradburn S., Murgatroyd C. (2018). In vivo cholinergic basal forebrain atrophy predicts cognitive decline in de novo Parkinson's disease. Brain.

[b0175] Reislev N.L., Kupers R., Siebner H.R., Ptito M., Dyrby T.B. (2016). Blindness alters the microstructure of the ventral but not the dorsal visual stream. Brain Struct. Func..

[b0180] Russell G., Harper R., Allen H., Baldwin R., Burns A. (2018). Cognitive impairment and Charles Bonnet syndrome: a prospective study. Int. J. Geriatr. Psychiatry.

[b0185] Santhouse A.M., Howard R.J., ffytche D.H. (2000). Visual hallucinatory syndromes and the anatomy of the visual brain. Brain.

[b0190] Schlerf J., Ivry R.B., Diedrichsen J. (2012). Encoding of sensory prediction errors in the human cerebellum. J. Neurosci..

[b0195] Schumacher J., Taylor J.-P., Hamilton C.A. (2021). In vivo nucleus basalis of Meynert degeneration in mild cognitive impairment with Lewy bodies. NeuroImage: Clin..

[b0200] Schwarz C.G., Reid R.I., Gunter J.L. (2014). Improved DTI registration allows voxel-based analysis that outperforms tract-based spatial statistics. NeuroImage.

[b0205] Shine J.M., Halliday G., Naismith S.L., Lewis S.J.G. (2011). Visual misperceptions and hallucinations in Parkinson's disease: dysfunction of attentional control networks?. Mov. Disord..

[b0210] Terao T.S.C. (2000). Charles Bonnet syndrome and dementia. Lancet.

[b0215] Teunisse R.J., Zitman F.G., Cruysberg J.R.M., Hoefnagels W.H.L., Verbeek A.L.M. (1996). Visual hallucinations in psychologically normal people: Charles Bonnet's syndrome. Lancet.

[b0220] Thier P., Markandy A. (2019). Role of the vermal cerebellum in visually guided eye movements and visual motion perception. Annu. Rev. Vis. Sci..

[b0225] Tsukada H., Fujii H., Aihara K., Tsuda I. (2015). Computational model of visual hallucination in dementia with Lewy bodies. Neural Netw..

[b0230] Urwyler P., Nef T., Muri R. (2016). Visual hallucinations in eye disease and Lewy body disease. Am. J. Geriatr. Psychiatry.

[b0235] van Es D.M., van der Zwaag W., Knapen T. (2019). Topographic maps of visual space in the human cerebellum. Curr. Biol..

[b0240] Warrington S., Bryant K., Khrapitchev A. (2020). XTRACT - Standardised protocols for automated tractography and connectivity blueprints in the human and macaque brain. NeuroImage.

[b0245] Watanabe H., Senda J., Kato S. (2013). Cortical and subcortical brain atrophy in Parkinson's disease with visual hallucination. Mov. Disord..

[b0250] Yoshimine S., Ogawa S., Horiguchi H. (2018). Age-related macular degeneration affects the optic radiation white matter projecting to locations of retinal damage. Brain Struct. Func..

[b0255] Yuki N., Yoshioka A., Mizuhara R., Kimura T. (2020). Visual hallucinations and inferior longitudinal fasciculus in Parkinson's disease. Brain Behav..

[b0260] Zarkali A., McColgan P., Leyland L.-A., Lees A.J., Rees G., Weil R.S. (2020). Fiber-specific white matter reductions in Parkinson hallucinations and visual dysfunction. Neurology.

[b0265] Zhang H., Avants B.B., Yushkevich P.A. (2007). The high-dimensional tensor-based DTI registration algorithm. IEEE Trans. Med. Imaging.

[b0270] Zorzi G., Poggiali D., Cecchin D., Cagnin A. (2021). The role of cerebellum in visual hallucinations: a metabolic point of view. A commentary on Lawn and ffytche. Cortex.

[b0275] Zorzi G., Thiebaut de Schotten M., Manara R., Bussè C., Corbetta M., Cagnin A. (2021). White matter abnormalities of right hemisphere attention networks contribute to visual hallucinations in dementia with Lewy bodies. Cortex.

