# Supplementary material for: Investigation of structural brain changes in Charles Bonnet Syndrome
Source: Neuroimage Clin. 2022 May 11;35:103041. doi: 10.1016/j.nicl.2022.103041 (PMC9118504; doi:10.1016/j.nicl.2022.103041)

Supplementary material for Firbank et al “Investigation of structural brain changes in Charles Bonnet Syndrome”

eTable 1 Details of eye disease participants

| VH Type | Primary VH Type | Complex Phenomenology | Primary Eye Disease | Secondary Eye Disease | Acuity | Years Eye Disease | Years CBS | Age | Gender |
| --- | --- | --- | --- | --- | --- | --- | --- | --- | --- |
| No VH | -- | -- | Other | MD | 0.28 | 4 | -- | 72 | Female |
| No VH | -- | -- | Cataracts | None | 0.66 | 0.5 | -- | 72 | Male |
| No VH | -- | -- | Glaucoma | MD | 0.39 | 0.5 | -- | 81 | Male |
| No VH | -- | -- | Other | None | 0.61 | 8 | -- | 71 | Female |
| No VH | -- | -- | MD | None | 0.1 | 0.5 | -- | 78 | Female |
| No VH | -- | -- | MD | None | 0.11 | 3 | -- | 68 | Female |
| No VH | -- | -- | MD | None | 0.62 | 6 | -- | 85 | Male |
| No VH | -- | -- | Other | None | 0.11 | 3 | -- | 67 | Male |
| No VH | -- | -- | MD | None | 0.15 | 6 | -- | 87 | Female |
| No VH | -- | -- | MD | None | 0.41 | 0.5 | -- | 85 | Female |
| No VH | -- | -- | MD | None | 0.17 | 2 | -- | 85 | Male |
| No VH | -- | -- | MD | Cataracts | 0.67 | 4 | -- | 73 | Male |
| No VH | -- | -- | MD | None | 0.68 | 3 | -- | 86 | Female |
| No VH | -- | -- | MD | None | 0.38 | 2 | -- | 86 | Female |
| No VH | -- | -- | MD | Glaucoma | 0.27 | 4 | -- | 75 | Female |
| No VH | -- | -- | MD | None | 0.56 | 34 | -- | 84 | Female |
| No VH | -- | -- | MD | None | 0.24 | 3 | -- | 77 | Female |
| Simple Only | Simple | -- | MD | Glaucoma | 0.89 | 4 | 1 | 73 | Female |
| Simple Only | Simple | -- | MD | None | 0.33 | 21 | 2 | 79 | Male |
| Simple Only | Simple | -- | Glaucoma | None | 0.0125 | 7 | 1 | 71 | Male |
| Simple Only | Simple | -- | Retinitis Pigmentosa | Glaucoma | 0.0125 | 61 | 10 | 68 | Male |
| Simple & Complex | Simple | Faces | MD | Cataracts | 0.28 | 5 | 3 | 92 | Female |
| Simple & Complex | Simple | Faces | Other | Cataracts | 0.69 | 3 | 2 | 53 | Female |
| Simple & Complex | Simple | Patterns | MD | None | 0.19 | 17 | 2 | 83 | Female |
| Simple & Complex | Complex | Patterns | MD | None | 0.21 | 15 | 5 | 75 | Male |
| Simple & Complex | Complex | Scenes | Other | None | 0.0125 | 13 | 13 | 75 | Female |
| Simple & Complex | Complex | Animals | MD | None | 0.85 | 2 | 1 | 80 | Female |
| Simple & Complex | Complex | Patterns | MD | None | 0.15 | 4 | 3 | 85 | Female |
| Simple & Complex | Complex | Objects | MD | Cataracts | 0.23 | 4 | 4 | 89 | Female |
| Simple & Complex | Complex | Patterns | MD | None | 0.18 | 1 | 1 | 85 | Female |
| Simple & Complex | Complex | Patterns | Glaucoma | None | 0.077 | 17 | 3 | 67 | Female |
| Complex Only | Complex | Patterns | MD | None | 0.16 | 6 | 1 | 93 | Female |
| Complex Only | Complex | Objects | MD | Glaucoma | 0.076 | -- | 4 | 86 | Male |

eTable 2 clusters of significantly reduced FA in the visually impaired group compared to the normally sighted controls. Labels are overlap with the JHU white matter tracts atlas JHU-ICBM-tracts-maxprob-thr0-1mm.nii and JHU WM labels.

| Cluster P | Cluster size(vox) | max_x | max_y | max_z | max_t stats | Region name |
| --- | --- | --- | --- | --- | --- | --- |
| <0.001 | 6565 | -12 | -3 | -4 | 9.16 | 52% 'Anterior thalamic radiation L' 3% 'Corticospinal tract L' 6% 'Inferior fronto-occipital fasciculus L' 22% 'Superior longitudinal fasciculus L' |
| <0.001 | 3476 | 10 | 2 | -2 | 6.66 | 77% 'Anterior thalamic radiation R' 5% 'Corticospinal tract R' |
| <0.001 | 2337 | 2 | 19 | 2 | 5.22 | 18% 'Cingulum (cingulate gyrus) L' 42% 'Forceps minor' |
| <0.001 | 1646 | -27 | -32 | 1 | 5.87 | 42% 'Anterior thalamic radiation L' 5% 'Corticospinal tract L' 8% 'Inferior longitudinal fasciculus L' 19% 'Inferior fronto-occipital fasciculus L'  26% 'Fornix (cres) / Stria terminalis L' |
| <0.001 | 1033 | -15 | -43 | 26 | 5.29 | 9% 'Anterior thalamic radiation L' 42% 'Cingulum (cingulate gyrus) L' 39% 'Superior longitudinal fasciculus L' |
| <0.001 | 963 | 36 | -1 | 28 | 6.09 | 83% 'Superior longitudinal fasciculus R' |
| <0.001 | 561 | 27 | -27 | -5 | 7.43 | 29% 'Anterior thalamic radiation R' 13% 'Inferior fronto-occipital fasciculus R'  53% 'Fornix (cres) / Stria terminalis R' |
| <0.001 | 377 | -12 | -30 | 52 | 4.66 | 40% 'Corticospinal tract L' 20% 'Cingulum (cingulate gyrus) L'  5% 'Anterior thalamic radiation L' |
| <0.001 | 332 | -45 | -28 | 0 | 5.36 | 20% 'Inferior longitudinal fasciculus L' 48% 'Superior longitudinal fasciculus L' |
| 0.001 | 299 | -26 | -17 | 46 | 4.82 | 11% 'Corticospinal tract L' 44% 'Superior longitudinal fasciculus L' |
| 0.001 | 292 | 10 | -7 | 31 | 5.15 | 8% 'Cingulum (cingulate gyrus) R'  97% ‘Body of Corpus Callosum’ |
| 0.003 | 252 | -17 | 46 | 17 | 5.76 | 13% 'Cingulum (cingulate gyrus) L' 87% 'Forceps minor' |
| 0.008 | 210 | -34 | 30 | 11 | 6.44 | 15% 'Anterior thalamic radiation L' 72% 'Inferior fronto-occipital fasciculus L' 8% 'Uncinate fasciculus L' |
| 0.009 | 207 | 22 | 31 | 24 | 4.97 | 44% 'Anterior thalamic radiation R' 40% 'Forceps minor' |
| 0.009 | 204 | 52 | -29 | 7 | 5.20 | 30% 'Inferior longitudinal fasciculus R' |
| 0.016 | 185 | -37 | -51 | -2 | 4.95 | 82% 'Inferior fronto-occipital fasciculus L' 18% 'Inferior longitudinal fasciculus L' |
| 0.031 | 163 | 28 | 17 | 8 | 4.39 | 47% 'Anterior thalamic radiation R' 51% 'Inferior fronto-occipital fasciculus R' |
| 0.034 | 160 | -42 | -42 | 33 | 5.22 | 100% 'Superior longitudinal fasciculus L' |
| 0.044 | 151 | 21 | -36 | 31 | 4.85 | 19% 'Anterior thalamic radiation R' 19% 'Cingulum (cingulate gyrus) R' |
| 0.048 | 148 | -30 | 28 | 22 | 4.56 | 60% 'Anterior thalamic radiation L' |

eTable 3 clusters of significantly increased MD in the visually impaired group compared to the normally sighted controls. Labels are overlap with the JHU white matter tracts atlas JHU-ICBM-tracts-maxprob-thr0-1mm.nii

| Cluster p | size | max_x | max_y | max_z | max_val | Region name |
| --- | --- | --- | --- | --- | --- | --- |
| <0.001 | 1898 | 11 | 0 | -6 | 8.37 | 50% 'Anterior thalamic radiation R' 5% 'Corticospinal tract R' 11% 'Inferior fronto-occipital fasciculus R' |
| <0.001 | 1741 | -11 | -17 | -4 | 6.74 | 34% 'Anterior thalamic radiation L' 52% 'Anterior thalamic radiation R' 5% 'Corticospinal tract R' |
| <0.001 | 1273 | -2 | -28 | -24 | 6.42 | 24% 'Anterior thalamic radiation L' 17% 'Anterior thalamic radiation R' 28% 'Corticospinal tract L' 9% 'Corticospinal tract R' |
| <0.001 | 1259 | -11 | 0 | -4 | 9.11 | 66% 'Anterior thalamic radiation L' |
| <0.001 | 850 | -44 | 3 | 13 | 4.86 | 98% 'Superior longitudinal fasciculus L' |
| <0.001 | 448 | -39 | 24 | 16 | 5.23 | 26% 'Anterior thalamic radiation L' 29% 'Inferior fronto-occipital fasciculus L' 19% 'Superior longitudinal fasciculus L' 7% 'Uncinate fasciculus L' |
| <0.001 | 446 | -27 | -27 | -2 | 6.69 | 40% 'Anterior thalamic radiation L' 5% 'Corticospinal tract L' 5% 'Inferior fronto-occipital fasciculus L' 10% 'Inferior longitudinal fasciculus L'  42% 'Fornix (cres) / Stria terminalis L' |
| 0.002 | 356 | -7 | 21 | 21 | 4.88 | 54% 'Cingulum (cingulate gyrus) L' 37% 'Forceps minor' |
| 0.006 | 292 | -18 | 15 | 7 | 4.36 | 83% 'Anterior thalamic radiation L' 14% 'Inferior fronto-occipital fasciculus L' |
| 0.013 | 252 | -7 | -13 | 12 | 5.54 | 100% 'Anterior thalamic radiation L' |
| 0.015 | 243 | -46 | -32 | 30 | 4.97 | 95% 'Superior longitudinal fasciculus L' |
| 0.027 | 217 | -25 | 8 | -11 | 4.87 | 28% 'Inferior fronto-occipital fasciculus L' 69% 'Uncinate fasciculus L' |
| 0.034 | 207 | -18 | -54 | -35 | 4.67 | 25% 'Anterior thalamic radiation L' 14% 'Corticospinal tract L' |

eTable 4 CBS vs Control-ED controlling for acuity and duration of eye disease

| ROI | CBS | Control_ED | CBS v Control-ED  P value | Age  P value | Gender  P value | Acuity  P value | Duration ED  P value | TIV  P value | Fstat |
| --- | --- | --- | --- | --- | --- | --- | --- | --- | --- |
|  | N = 16 | N = 17 |  |  |  |  |  |  |  |
| TBV (litres) | 0.937 (0.13) | 0.92 (0.081) | 0.581 | <0.001* | 0.596 | 0.575 | 0.668 | <0.001* | F_6,25_ = 31.01 |
| Occipital pole | 3173.8 (1065.2) | 3179.4 (374.2) | 0.606 | 0.245 | 0.353 | 0.229 | 0.790 | <0.001* | F_6,25_ = 8.89 |
| V1_ant | 7073.1 (1187.1) | 7093.8 (835.9) | 0.660 | <0.001* | 0.016 * | 0.03* | 0.719 | 0.185 | F_6,25_ = 7.35 |
| V2_ant | 3421 (489.9) | 3449.7 (333.6) | 0.458 | 0.005 * | 0.053 | 0.046* | 0.614 | 0.050 * | F_6,25_ = 3.85 |
| V3a | 2001.8 (414.3) | 1959.3 (284.7) | 0.706 | 0.413 | 0.336 | 0.904 | 0.525 | 0.183 | F_6,25_ = 2.53 |
| V3d | 3516.5 (735.7) | 3536.9 (450.8) | 0.810 | 0.281 | 0.827 | 0.577 | 0.930 | 0.027 * | F_6,25_ = 2.84 |
| V3v | 4288.2 (828.7) | 4418.4 (423.7) | 0.279 | 0.001 * | 0.480 | 0.019* | 0.393 | 0.008 * | F_6,25_ = 8.16 |
| V4v | 3757.1 (682.3) | 3884.8 (380) | 0.500 | 0.005 * | 0.763 | 0.195 | 0.640 | 0.270 | F_6,25_ = 3.87 |
| hOc4la | 5189.5 (962.5) | 5297.7 (533.8) | 0.332 | 0.009 * | 0.822 | 0.856 | 0.251 | 0.011 * | F_6,25_ = 7.61 |
| hOc4lp | 3718.5 (802.6) | 3936 (460.7) | 0.260 | 0.006 * | 0.427 | 0.847 | 0.576 | 0.066 | F_6,25_ = 6.01 |
| V5 | 504 (129.6) | 519.2 (82.6) | 0.417 | 0.013 * | 0.894 | 0.128 | 0.030 * | 0.712 | F_6,25_ = 3.21 |
| Hippocampus | 4426.2 (805.4) | 4162.1 (564.9) | 0.426 | <0.001 * | 0.012 * | 0.384 | 0.520 | <0.001 * | F_6,25_ = 7.53 |
| nBM | 221 (37.8) | 216.7 (23.9) | 0.727 | 0.002* | 0.117 | 0.118 | 0.419 | 0.009 * | F_6,25_ = 5.38 |

TIV = total intracranial volume. nBM = nucleus Basalis of Meynert. Occipital pole is the intersection of regions V1 and V2 with the neuromorphic atlas occipital pole region. V1_ant and V2_ant are the V1 and V2 regions excluding the occipital pole area.

eTable 5 Regression within the eye disease group controlling for visual acuity and duration of eye disease.

| ROI | Patient | Control_ED | CBS v Control_ED  P value | Age  P value | Gender  P value | Acuity  P value | Duration ED  P value | TIV  P value | Fstat |
| --- | --- | --- | --- | --- | --- | --- | --- | --- | --- |
|  | N = 16 | N = 17 |  |  |  |  |  |  |  |
| ILF mean FA | 0.331 (0.019) | 0.334 (0.019) | 0.762 | 0.067 | 0.624 | 0.721 | 0.015 * | - | F_5,26_ = 2.15 |
| OR mean FA | 0.364 (0.024) | 0.369 (0.025) | 0.752 | 0.208 | 0.309 | 0.215 | 0.124 | - | F_5,26_ = 1.52 |
| VOF mean FA | 0.264 (0.021) | 0.272 (0.019) | 0.888 | 0.466 | 0.612 | 0.474 | 0.019 * | - | F_5,26_ = 1.88 |
| IFOF mean FA | 0.335 (0.02) | 0.339 (0.019) | 0.799 | 0.126 | 0.718 | 0.385 | 0.096 | - | F_5,26_ = 1.39 |
| SLF3 mean FA | 0.282 (0.016) | 0.283 (0.015) | 0.761 | 0.772 | 0.868 | 0.546 | 0.587 | - | F_5,26_ = 0.2 |
| ILF mean MD | 892 (36) | 891 (44) | 0.828 | 0.009 * | 0.415 | 0.492 | 0.203 | - | F_5,26_ = 2.16 |
| OR mean MD | 940 (49) | 945 (54) | 0.370 | 0.014 * | 0.787 | 0.369 | 0.179 | - | F_5,26_ = 2.08 |
| VOF mean MD | 1019 (66) | 1005 (63) | 0.859 | 0.080 | 0.876 | 0.276 | 0.031 * | - | F_5,26_ = 2.21 |
| IFOF mean MD | 925 (41) | 924 (49) | 0.735 | 0.003 * | 0.566 | 0.527 | 0.334 | - | F_5,26_ = 2.41 |
| SLF3 mean MD | 1048 (57) | 1046 (48) | 0.778 | 0.114 | 0.083 | 0.169 | 0.792 | - | F_5,26_ = 1.66 |
| ILF volume mm^3^ | 29689 (3370) | 28333 (3400) | 0.412 | 0.731 | 0.136 | 0.845 | 0.300 | 0.001 * | F_6,25_ = 3.17 |
| OR volume mm^3^ | 29902 (3876) | 29560 (2477) | 0.791 | 0.547 | 0.958 | 0.338 | 0.438 | 0.045 * | F_6,25_ = 2.59 |
| VOF volume mm^3^ | 18256 (3069) | 18524 (2588) | 0.463 | 0.668 | 0.098 | 0.390 | 0.721 | 0.005 * | F_6,25_ = 2 |
| IFOF volume mm^3^ | 50040 (6290) | 49232 (5366) | 0.725 | 0.708 | 0.783 | 0.474 | 0.654 | 0.019 * | F_6,25_ = 3.36 |
| SLF3 volume mm^3^ | 28055 (4767) | 28903 (5597) | 0.501 | 0.420 | 0.905 | 0.542 | 0.690 | 0.243 | F_6,25_ = 0.75 |

ILF = inferior longitudinal fasciculus , OR = optic radiation, VOF = vertical occipital fasciculus, IFOF = inferior fronto-occipital fasciculus, SLF3 = superior longitudinal fasciculus3, TIV = total intracranial volume.

eTable 6 Structural differences in complex vs simple hallucinations controlling for actuity and duration of eye disease

| ROI | Simple | Complex | Simple vs complex  P value | Age  P value | Gender  P value | Acuity  P value | Duration ED  P value | TIV p value | Fstat |
| --- | --- | --- | --- | --- | --- | --- | --- | --- | --- |
|  | N = 7 | N = 9 |  |  |  |  |  |  |  |
| TBV (litres) | 0.966 (0.121) | 0.914 (0.139) | 0.581 | 0.003 * | 0.341 | 0.656 | 0.220 | 0.005 * | F_6,8_ = 23.8 |
| Occipital pole | 3576 (955.5) | 2861 (1091.3) | 0.199 | 0.728 | 0.734 | 0.154 | 0.615 | 0.020 * | F_6,8_ = 9.4 |
| V1_ant | 7530.8 (1328.3) | 6717 (996.4) | 0.298 | 0.003 * | 0.770 | 0.011 * | 0.230 | 0.596 | F_6,8_ = 10.33 |
| V2_ant | 3684.7 (426.9) | 3216 (453) | 0.154 | 0.149 | 0.193 | 0.059 | 0.219 | 0.994 | F_6,8_ = 5.36 |
| V3a | 2096.1 (326.4) | 1928.4 (477.6) | 0.864 | 0.647 | 0.082 | 0.698 | 0.439 | 0.884 | F_6,8_ = 2.99 |
| V3d | 3853 (456.9) | 3254.8 (825.9) | 0.496 | 0.890 | 0.156 | 0.500 | 0.607 | 0.691 | F_6,8_ = 3.71 |
| V3v | 4758.8 (643.1) | 3922.2 (795.6) | 0.169 | 0.025 * | 0.148 | 0.121 | 0.274 | 0.479 | F_6,8_ = 9.52 |
| V4v | 4108.1 (631.9) | 3484.2 (617.9) | 0.689 | 0.086 | 0.085 | 0.339 | 0.346 | 0.631 | F_6,8_ = 3.89 |
| hOc4la | 5682.3 (1067.9) | 4806.2 (709.8) | 0.307 | 0.095 | 0.036 * | 0.887 | 0.358 | 0.651 | F_6,8_ = 10.85 |
| hOc4lp | 4106.9 (798.6) | 3416.4 (703.4) | 0.292 | 0.059 | 0.136 | 0.473 | 0.155 | 0.811 | F_6,8_ = 5.31 |
| V5 | 583.9 (137.9) | 441.9 (85.4) | 0.486 | 0.344 | 0.602 | 0.284 | 0.290 | 0.783 | F_6,8_ = 2.04 |
| Hippocampus | 4602.3 (831.8) | 4289.2 (805.7) | 0.715 | 0.072 | 0.424 | 0.684 | 0.313 | 0.209 | F_6,8_ = 4.75 |
| nBM | 218.9 (33.4) | 222.6 (42.8) | 0.451 | 0.009 * | 0.355 | 0.682 | 0.861 | 0.043 * | F_6,8_ = 4.94 |

TIV = total intracranial volume. nBM = nucleus Basalis of Meynert. Occipital pole is the intersection of regions V1 and V2 with the neuromorphic atlas occipital pole region. V1_ant and V2_ant are the V1 and V2 regions excluding the occipital pole area.

eTable 7 White matter tract difference in complex vs simple CBS patients controlling for acuity and duration of eye disease

| ROI | Simple | Complex | Simple vs complex  P value | Age  P value | Gender  P value | Acuity  P value | Duration ED  P value | TIV  P value | Fstat |
| --- | --- | --- | --- | --- | --- | --- | --- | --- | --- |
|  | N = 7 | N = 9 |  |  |  |  |  |  |  |
| ILF mean FA | 0.329 (0.023) | 0.333 (0.017) | 0.727 | 0.527 | 0.369 | 0.557 | 0.141 | - | F_5,9_ = 1.01 |
| OR mean FA | 0.364 (0.027) | 0.365 (0.024) | 0.767 | 0.566 | 0.372 | 0.615 | 0.244 | - | F_5,9_ = 0.64 |
| VOF mean FA | 0.262 (0.027) | 0.266 (0.016) | 0.856 | 0.424 | 0.529 | 0.955 | 0.082 | - | F_5,9_ = 1.04 |
| IFOF mean FA | 0.334 (0.024) | 0.335 (0.018) | 0.730 | 0.540 | 0.504 | 0.486 | 0.278 | - | F_5,9_ = 0.72 |
| SLF3 mean FA | 0.278 (0.016) | 0.286 (0.015) | 0.585 | 0.074 | 0.6 | 0.278 | 0.097 | - | F_5,9_ = 1.7 |
| ILF mean MD | 897 (44) | 888 (31) | 0.326 | 0.033 * | 0.818 | 0.306 | 0.358 | - | F_5,9_ = 2.58 |
| OR mean MD | 940 (50) | 940 (51) | 0.629 | 0.028 * | 0.783 | 0.933 | 0.136 | - | F_5,9_ = 1.99 |
| VOF mean MD | 1016 (81) | 1021 (57) | 0.962 | 0.105 | 0.707 | 0.693 | 0.156 | - | F_5,9_ = 1.44 |
| IFOF mean MD | 925 (51) | 925 (35) | 0.405 | 0.029 * | 0.871 | 0.291 | 0.464 | - | F_5,9_ = 2.48 |
| SLF3 mean MD | 1047 (71) | 1049 (49) | 0.762 | 0.006 * | 0.113 | 0.922 | 0.214 | - | F_5,9_ = 4.32 |
| ILF volume mm^3^ | 30151 (2502) | 29329 (4033) | 0.240 | 0.251 | 0.62 | 0.895 | 0.625 | 0.094 | F_6,8_ = 1.83 |
| OR volume mm^3^ | 31107 (4958) | 28965 (2734) | 0.378 | 0.425 | 0.714 | 0.732 | 0.870 | 0.353 | F_6,8_ = 1.79 |
| VOF volume mm^3^ | 17962 (3754) | 18484 (2637) | 0.861 | 0.694 | 0.548 | 0.383 | 0.227 | 0.435 | F_6,8_ = 1.72 |
| IFOF volume mm^3^ | 50387 (6836) | 49770 (6240) | 0.944 | 0.515 | 0.586 | 0.703 | 0.685 | 0.361 | F_6,8_ = 1.77 |
| SLF3 volume mm^3^ | 27737 (6404) | 28302 (3419) | 0.573 | 0.600 | 0.51 | 0.890 | 0.783 | 0.593 | F_6,8_ = 1.16 |

ILF = inferior longitudinal fasciculus , OR = optic radiation, VOF = vertical occipital fasciculus, IFOF = inferior fronto-occipital fasciculus, SLF3 = superior longitudinal fasciculus3, TIV = total intracranial volume.

eTable 8 Clusters of reduced cerebellar grey matter density in the simple vs complex hallucinators

| Cluster p | size | max_x | max_y | max_z | max_val | Region name |
| --- | --- | --- | --- | --- | --- | --- |
| <0.001 | 109 | 18 | -84 | -41 | 6.355383 | 76% Right Lobule VIIa Crus II  9% Right Lobule VIIb  7% Right Lobule VIIA Crus I |
| 0.00941 | 41 | -24 | -50 | -59 | 4.976377 | Left Lobule VIIIb |
| 0.05634 | 28 | 16 | -54 | -55 | 4.96431 | 75% Right Lobule VIIIb  25% Right Lobule IX |

eFigure 1 Occipital regions of interest used. hOc5 light blue; hOc4v dark blue; hOc4lp pink; hOc4la yellow; hOc4d green; hOc3v orange; hOc3d brown; hOc2 dark grey; hOc1 red; occipital pole V1/V2 white


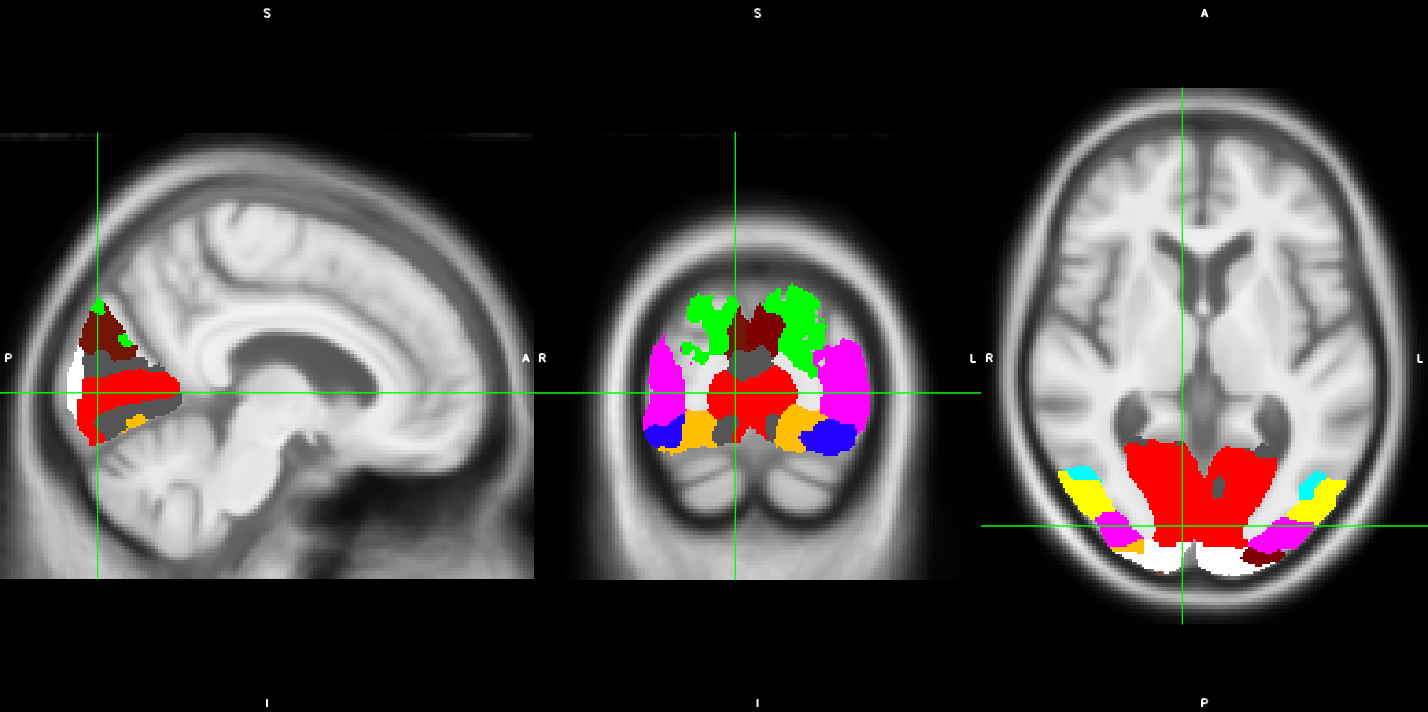


eFigure 2 Mean FA in the optic radiation and anterior thalamic radiation vs. duration of eye disease


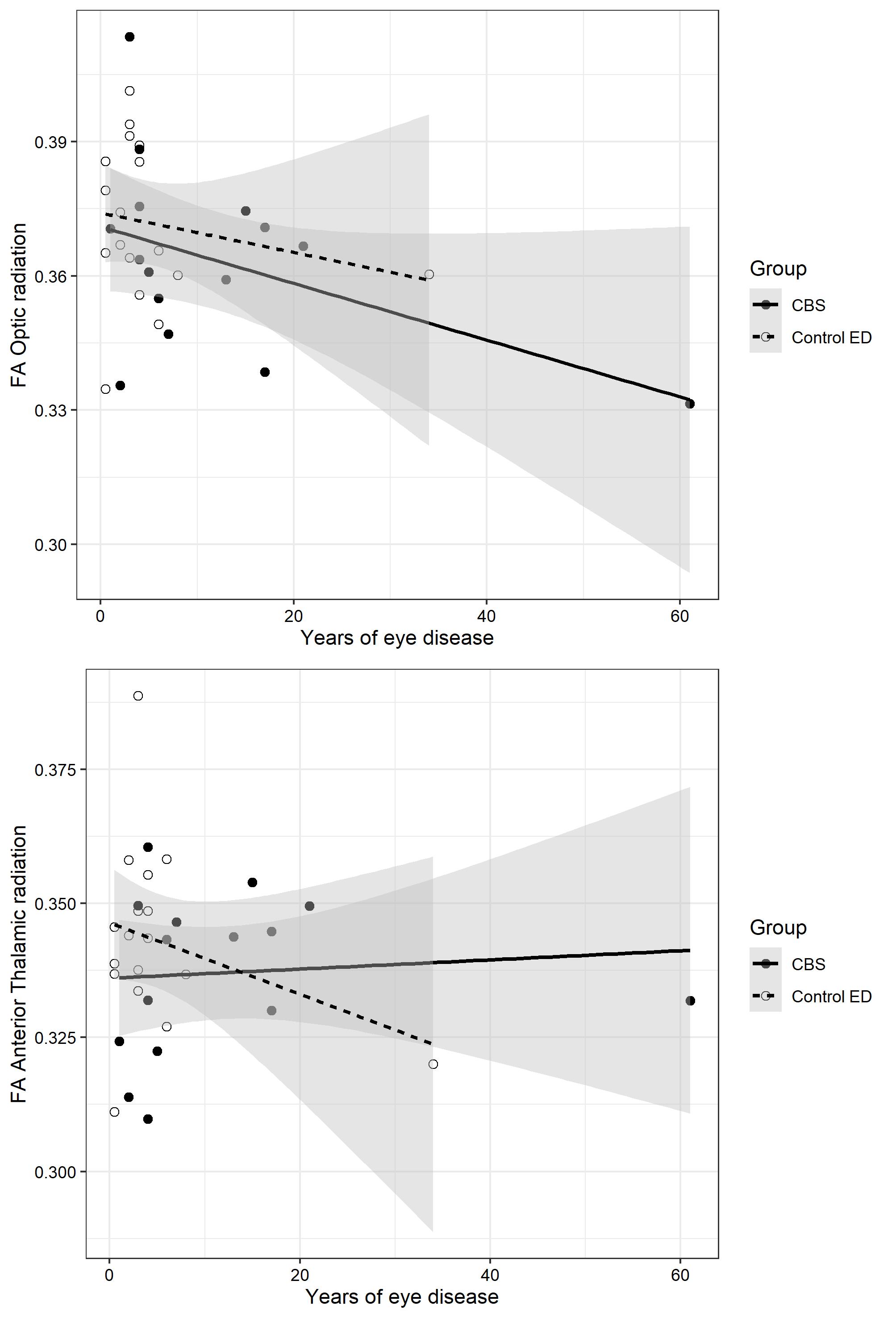


eFigure 3

Visual Hallucination NPI Frequency x Severity score vs Hippocampus volume with effects of age, sex and intracranial volume regressed out.


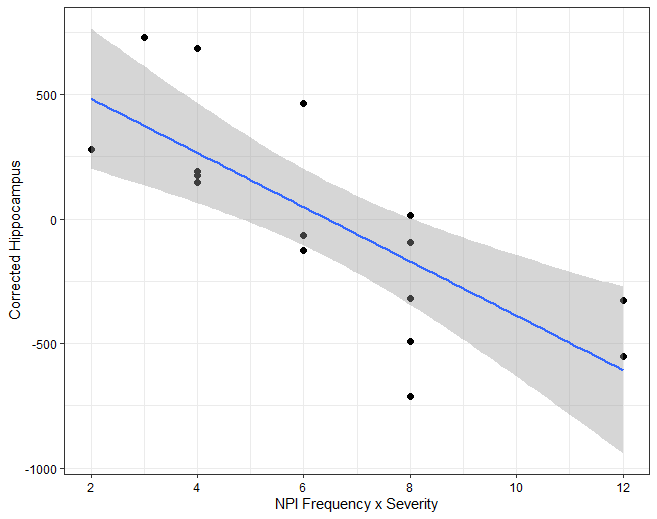


eFigure 4 Cerebellar lobule VIIIb in eye disease


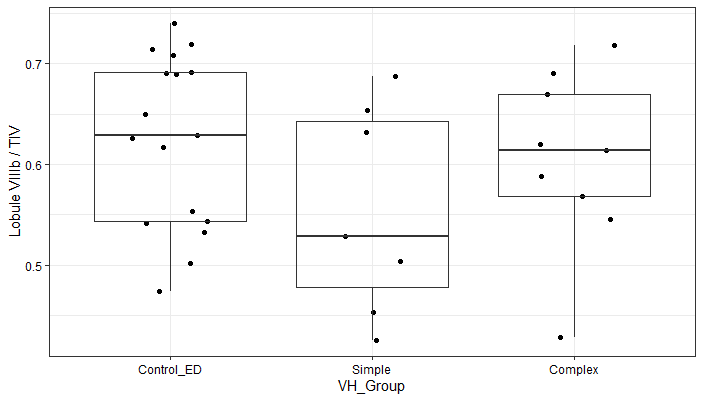

Supplement: Supplementary data 1 [file mmc1.docx]
